# Spider vs. guns: expectancy and attention biases to phylogenetic threat do not extend to ontogenetic threat

**DOI:** 10.3389/fpsyg.2023.1232985

**Published:** 2023-08-30

**Authors:** Elinor Abado, Tatjana Aue, Hadas Okon-Singer

**Affiliations:** ^1^School of Psychological Sciences, University of Haifa, Haifa, Israel; ^2^The Integrated Brain and Behavior Research Center (IBBRC), University of Haifa, Haifa, Israel; ^3^Institute of Psychology, University of Bern, Bern, Switzerland

**Keywords:** attention bias, expectancy bias, phylogenetic threat, ontogenetic threat, specific fear

## Abstract

**Introduction:**

Attention bias plays an important role in specific fears and phobias. Previous studies revealed that *a-priori* expectancies affect attention toward neutral stimuli but not threatening stimuli. The aim of the current study was to test whether this selective influence of expectancies on attention is specific to phylogenetic threat (i.e., spiders) or whether it can be generalized to ontogenetic threat (i.e., guns). Correspondingly, we directly compared expectancy effects on attentional allocation to phylogenetically vs. ontogenetically threatening stimuli.

**Method:**

Expectancies were manipulated by presenting a cue indicating the likelihood of the appearance of a deviant picture in a visual search array. The array included eight distractors and one neutral (phone/bird) or threatening (gun/spider) deviant picture. In a comprehensive design, we examined the effects of stimulus type (phylogenetic/ontogenetic) and visual background (white and sterile/complex and ecological). Individual differences such as intolerance of uncertainty and spider fear were also measured.

**Results:**

Results showed that attention bias toward spiders does not extend to threatening ontogenetic stimuli (i.e., guns). Our previous findings on attention bias toward spiders were replicated and a small to medium positive correlation was found between reaction time to bird targets and pre-existing fear of spider levels. Cues were used to detect threatening as well as neutral targets on both background types, except for spider targets on a complex background, replicating previous results. A small to medium positive correlation was also found between fear of spiders and intolerance of uncertainty.

**Discussion:**

Together, these results suggest that expectancy and attentional processes may differ between ontogenetic and phylogenetic threat. Importantly, the effects of expectancy on attentional allocation depend on an interaction between the type of threat (ontogenetic/phylogenetic), visual factors, and individual differences.

## Introduction

### Cognitive biases and spider fear and anxiety

Anxiety disorders are characterized by cognitive biases exhibited toward anxiety-relevant stimuli. For instance, individuals with spider phobia can exhibit selective attention toward spiders (e.g., Öhman et al., 2001), overestimate the risk of encountering them (e.g., Aue and Hoeppli, 2012), and even misinterpret beetles as spiders (e.g., Becker and Rinck, 2004). Cognitive biases can also be found in healthy populations, as they, too, often find anxiety- and fear-relevant stimuli aversive (Aue and Okon-Singer, 2020). The present study focused on two well-established biases: attention bias and expectancy bias. *Attention bias* reflects faster engagement with feared than with neutral stimuli (Cisler and Koster, 2010; Okon-Singer, 2018; Abado et al., 2020b). *Expectancy (encounter) bias* reflects the overestimation of the likelihood of encountering the fearful stimulus.

Only a few studies investigated the interplay between expectancy bias and attention bias. These studies examined the interaction of the two biases in individuals with spider phobia and in individuals without spider phobia (Aue et al., 2013, 2016, 2019; Abado et al., 2020c). In these studies, expectancy bias was manipulated using a verbal cue indicating the likelihood of the appearance of a certain target stimulus in the following visual search array. These cues included a spider cue (“spider 90%”), a neutral cue (“bird 90%”) and an ambiguous cue (“spider-bird 50%”). Following the cue, a visual search array was presented. The array included one target: either a bird or a spider, which appeared among pictures of non-threatening distractors (butterflies). As expected, participants exhibited a general attention bias toward spider targets by detecting them faster than bird targets. Interestingly, an interaction was found between cue and target, as cues had an impact on the detection of bird targets, while the detection of spider targets was unaffected by the cues (Aue et al., 2013, 2016, 2019). These findings suggest that attention deployment to spiders appears somewhat impenetrable to *a-priori* expectancies.

Previous studies have suggested that attention bias toward spiders exists for evolutionary reasons (Seligman, 1971; see also Coelho et al., 2019 for a recent discussion on alternative theories). According to the biological preparedness hypothesis, avoidance of spiders may be considered an adaptive behavior, as it is found in healthy populations as well – sometimes to a lesser and sometimes to an equal extent compared with participants with phobia (Aue and Okon-Singer, 2020). This argument further receives support from studies showing it is difficult to extinguish fear toward phylogenetic threat (Seligman, 1971; for reviews, see Marks and Nesse, 1994; Öhman and Mineka, 2001).

Ontogenetic threat describes threatening stimuli that are based on socio-cultural learning instead of being rooted in human evolution. More recent investigations suggest similar extinction processes for both phylogenetic and ontogenetic threatening stimuli (e.g., Flykt et al., 2007; Luck et al., 2020). Thus, the debate on whether humans are predisposed to be afraid of certain stimuli and whether this fear is indeed particularly difficult to extinguish, is still ongoing (for a recent systematic review which suggests that there is not enough evidence to support the biological preparedness hypothesis, see Åhs et al., 2018; for a recent commentary which suggests that there is indeed enough evidence to support the biological preparedness hypothesis, see Del Giudice, 2021).

In order to examine the role of evolutionary considerations in cognitive biases to threat, several studies compared phylogenetic threat (i.e., evolutionary relevant, such as spiders and snakes) with ontogenetic threat (i.e., modern threat, such as guns and knives). In the case of biased expectancies, for instance, studies examined participants’ *a-priori* expectancies regarding the pairing of the presentation of different kinds of stimuli (e.g., spiders and guns) with different kinds of outcomes (e.g., electric shock and neutral sound). Such studies also measured participants’ post-experimental estimation of how often a certain stimulus was paired with a certain outcome during the experiment. In reality, the pairings between each stimulus and outcome are equally distributed, wherefore no bias in favor of negative outcomes for spiders should arise. For instance, Mühlberger et al. (2006) measured *a-priori* and *a posteriori* covariation bias in participants with spider phobia or flight phobia. For *a-priori* estimates, results showed that each fear group exhibited expectancy bias for its disorder-specific threat. However, post-experimental disorder-specific covariation bias emerged only in the spider phobia group and not in the flight phobia group. Overall, studies on expectancy/covariation biases toward phylogenetic and ontogenetic threat yield mixed results and suggest that several factors may affect bias toward any type of threat, such as pre-existing fear levels and methodological considerations (for a review on covariation biases toward ontogenetic and phylogenetic threat, see Wiemer and Pauli, 2016; see also Muris et al., 2005, 2007, for differing results on expectancy bias in phylogenetic vs. ontogenetic stimuli).

Studies that examined attention bias toward phylogenetic and ontogenetic threat also yielded mixed results. For instance, some studies found that even simple, abstract and schematic pictures of phylogenetically threatening animals can quickly capture attention and cause interference in performance (e.g., Forbes et al., 2011; LoBue, 2014; New and German, 2015). Other investigations revealed that ontogenetic threat is detected faster than phylogenetic threat and that event-related potentials (ERPs) differentiate between threatening and neutral ontogenetic stimuli but not between threatening and neutral phylogenetic stimuli (Cinq-Mars et al., 2022; see also Subra et al., 2018, for similar behavioral results in a paradigm in which threatening pictures were used as cues, not as targets). While some studies point to the possibility of different mechanisms underlying the processing of phylogenetic vs. ontogenetic threat, other studies found comparable processing of phylogenetic and ontogenetic threat. These inconsistencies have led to the suggestion that the determining factor of attention bias is the potential danger that could be posed by a stimulus, regardless of its evolutionary relevance (e.g., Brosch and Sharma, 2005).

Recently, Zsido et al. (2019a) compared attention processing between phylogenetic and ontogenetic stimuli in ecological contexts. Specifically, phylogenetic and ontogenetic stimuli were presented on forest backgrounds or on street backgrounds. Participants were asked to find different targets. In Experiment 1, participants were asked to detect exemplars of each type of stimulus (e.g., snakes and cats as phylogenetic threatening and neutral stimuli, respectively, and guns and pens as ontogenetic threatening and neutral stimuli, respectively). Results showed that all types of threatening stimuli were found more quickly than neutral stimuli, regardless of evolutionary relevance. In Experiment 2, more exemplars were added, and neutral targets were found faster on mismatched trials (i.e., evolutionary relevant targets on modern backgrounds or vice versa). These results suggest that visual contexts play a role in attentional deployment.

To summarize, mixed results exist regarding expectancies and attention bias toward ontogenetic vs. phylogenetic stimuli. While some studies found prioritized processing of phylogenetic threat, others found prioritized processing of ontogenetic threat and still other studies found comparable effects for both types of stimuli. Several factors have been suggested to affect the processing of threat, such as pre-existing fear levels and experimental manipulations (e.g., background type, type of expected outcome). It remains unknown how *a-priori* expectancies and attention interact in phylogenetic vs. ontogenetic stimuli and whether this interaction is affected by visual factors or by individual traits.

### The current study

In the current study, we aimed to directly compare the effects of expectancy on attention bias between ontogenetic stimuli and phylogenetic stimuli. Thus, attention bias was examined in two ways: by comparing attentional allocation toward threatening vs. neutral stimuli and by examining the effects of *a-priori* expectancy cues on attention allocation toward each stimulus type (threatening and neutral phylogenetic and ontogenetic stimuli). To this end, we used the same paradigm as in our previous studies (e.g., Aue et al., 2013, 2016, 2019) to test ontogenetic (i.e., guns) and phylogenetic (i.e., spiders) stimuli. We examined whether unselected participants react faster to guns/spiders than to non-threatening targets (i.e., old mobile phones/birds), and whether participants use expectancy cues in order to detect each target.

In order to control for potential visual confounds, each type of stimulus was presented on a different background: a white background or a natural background (e.g., spider on a leaf/tree, a gun in a hand). Subjective valence ratings were included at the end of the experiment to validate feelings of pleasantness and unpleasantness toward each type of stimulus. Individual differences were also measured, including fear of spiders, state anxiety and depression, as well as feelings of perceived uncontrollability, unpredictability, danger, and disgust toward spiders. Intolerance of uncertainty (IU) was also measured, as it has been found to a play critical role in anxiety disorders (for reviews, see Grupe and Nitschke, 2013; Abado et al., 2020b). However, its role in cognitive biases and specific fears remains understudied. Lastly, due to inconsistent reliability and within-subject differences in cognitive tasks (Hedge et al., 2018; Parsons et al., 2019), particularly in attention bias toward threat (Rodebaugh et al., 2016), split-half reliability analyses for attention bias were also included.

In the phylogenetic conditions, we expected participants to respond faster to spider targets compared to bird targets, regardless of the type of background. In line with our previous studies (Aue et al., 2013, 2016, 2019), we also expected participants to use cues to detect only bird targets, and not spider targets, on both background types. Due to the mixed effects found in the literature, we did not have specific hypotheses for the ontogenetic conditions. If participants exhibit a similar attention bias to phylogenetic as well as ontogenetic stimuli, we can conclude that evolutionary relevance is not the (sole) determinant of fear responses toward spiders. However, if attention toward ontogenetic threat is affected by expectancies, unlike attention toward phylogenetic threat, then a tentative case can be made for prioritized processing that is specific for phylogenetic stimuli.

## Method

### Participants

This experiment was approved by the ethics committee of the School of Psychological Sciences at the University of Haifa (approval #341/19). Sample size was determined using G*Power (version 3.1.9.4; Faul et al., 2007), with a medium effect size (0.06) and using the “as in SPSS” setting (see Miles and Shevlin, 2001; Cohen, 2013, for more on effect sizes). The calculation was based on the planned main analysis of two within-subject factors (*cue*: threatening, neutral, ambiguous; and *target*: threatening, neutral – overall 6 within-subject conditions), and two between-subject factors (*stimulus type*: phylogenetic, ontogenetic; and *background type*: white, complex – overall four between-subject groups). Accordingly, 108 participants (27 in each fear group) were needed to reach a power of 0.95 with an error probability of 0.02. Forty participants were recruited per group (160 overall), to counterbalance versions and to account for excluded participants (see exclusion criteria below). Participants were recruited online, using the Prolific Platform.^1^

Inclusion criteria consisted of normal or corrected-to normal vision. Exclusion criteria included a history of neurological disorders or ADHD. As participants with neurological history or ADHD could not be screened in advance on Prolific, participants who indicated neurological history or ADHD history during their participation were excluded post-experimentally. Participants were also excluded post-experimentally if they received a standard (Z) score in either dependent measure (RT or accuracy) that was larger than 2.5 in absolute terms.

Of the 160 participants, 16 were excluded from analysis: One reported a history of neurological disorders, five reported a history of ADHD, and ten were excluded due to slow responses or low accuracy rates, leaving 144 participants in the final analysis. Thus, 34 participants remained in the ontogenetic-white background condition (20 males, mean age = 22.53 ± 3.48), 36 participants in the ontogenetic-complex background condition (24 males; mean age = 26.92 ± 9.28) and in the phylogenetic-white background condition (23 males; mean age = 26.36 ± 8.47) and 38 participants in the phylogenetic-complex background condition (22 males; mean age = 24.92 ± 8.49).

### Materials

Before beginning the experiment, participants were asked to fill out the following questionnaires:

1. The Intolerance of Uncertainty Scale (IUS-12) – short form (Carleton et al., 2007): the short form includes 12 items on a five-point scale, ranging from 1 (“not at all characteristic of me”) to 5 (“entirely characteristic of me”). Examples of items include: “Unforeseen events upset me greatly” and “Uncertainty keeps me from living a full life.” The final score is equal to the summation of all items, so that higher scores indicate higher levels of IU. IU is a transdiagnostic trait, which has been found to correlate with many disorders and individual traits, especially generalized anxiety disorder (for a review, see Einstein, 2014; for a meta-analysis, see McEvoy et al., 2019).
2. State–Trait Anxiety Inventory (STAI; Spielberger, 2010): we used the state anxiety subscale of the inventory, which contains 20 questions and refers to state anxiety, i.e., how the participant is feeling at the moment of answering the questionnaire (e.g., “I am tense,” “I feel calm”). Each item is rated on a 4-point scale (e.g., from “1 – almost never” to “4 – almost always”). The final score is equal to the summation of all items, so that higher scores indicate higher levels of anxiety. The STAI shows high internal consistency (coefficients range from 0.86 to 0.95), as well as high test–retest reliability (0.65 to 0.75).
3. Beck Depression Inventory (BDI; Beck, 1991): the questionnaire contains 21 items. Each item represents a symptom of depression and is rated on a 4-point scale, from 0 to 3. For instance, the first item addresses sadness, and the scale is: “0. I do not feel sad,” “1. I feel sad,” “2. I am sad all the time and I cannot snap out of it,” and “3. I am so sad and unhappy that I cannot stand it.” The final score is equal to the summation of all items, so that higher scores indicate higher levels of depression. The BDI shows high internal consistency (alpha coefficients range from 0.82 to 0.88).

Following the experiment, participants were asked to fill the Fear of Spiders Questionnaire (FSQ; Szymanski and O’Donohue, 1995): the FSQ reliably differentiates between individuals with and without spider phobia. It contains 18 items, each rated on a seven-point Likert-scale, ranging from 1 to 7. Examples of items include: “If I came across a spider now, I would get help from someone else to remove it” and “If I saw a spider now, I would think it will harm me.” The total score equals the summation of all items, as higher scores indicate higher fear levels. The questionnaire shows high internal consistency (Cronbach’s alpha = 0.92; Szymanski and O’Donohue, 1995).

Lastly, participants were asked about perceived disgust, danger, uncontrollability, and unpredictability of spiders (Arntz et al., 1993; Armfield and Mattiske, 1996). The latter 4 dimension were rated on a Likert scale ranging from 1 to 7.

### Stimuli

For each condition, 30 threatening target pictures (i.e., guns or spiders), 30 neutral target pictures (i.e., phones or birds) and 100 neutral distractor pictures (i.e., staplers or butterflies) were collected. Pictures were matched for contrast and luminance using MATLAB (MathWorks; version 2017b; all *p*s > 0.05).

Pictures of phylogenetic stimuli on a complex background were the exact same pictures that were used in previous studies (Aue et al., 2013, 2016, 2019; pictures taken from Dan-Glauser and Scherer, 2011). Pictures for all other conditions were collected from the Internet. Pictures of ontogenetic stimuli included guns as threatening targets, old mobile phones as neutral targets, and staplers as distractors. Old mobile phones and staplers were chosen based on their similarity to guns as heavy, thick objects, so as to limit differentiating visual factors. Mobile phones have been used previously in similar experiments featuring visual search arrays containing guns (Brosch and Sharma, 2005; Zsido et al., 2019b), and office supplies have been shown to be neutral (Kurdi et al., 2017). For the ontogenetic stimuli on a complex background condition, four pictures were taken from the International Affective Picture System (Bradley and Lang, 2017) and one picture from the Open Affective Standardized Imaged Set (Kurdi et al., 2017). White background pictures included only the specific object/animal on a white background, while complex background pictures included animals in nature (e.g., on a tree) and objects in realistic settings (e.g., in a hand, on a desk, etc.).

### Procedure

**Figure 1:**
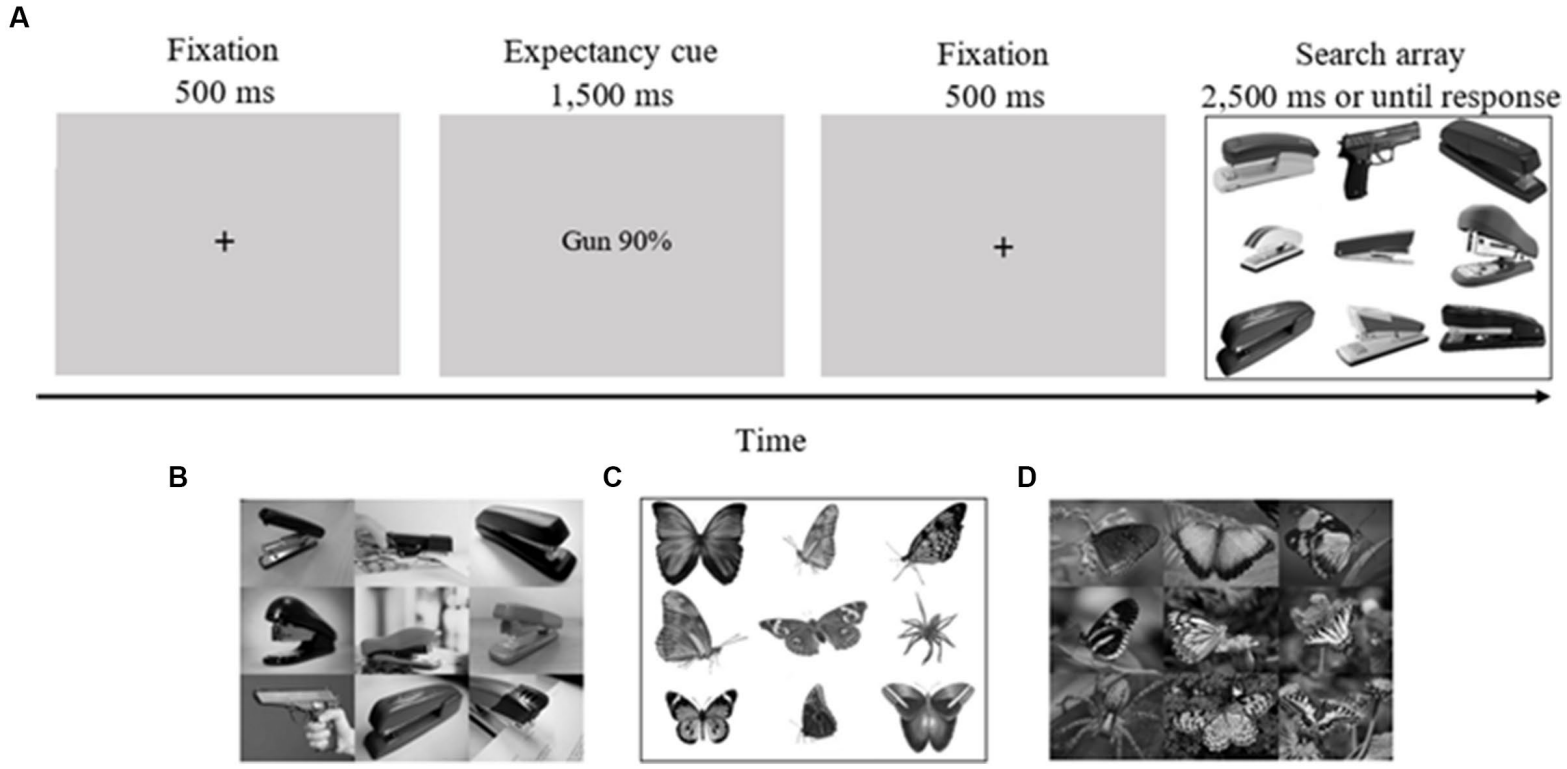
Task sequence of the experimental task. **(A)** An example of a valid trial for the guns on white background condition (gun in upper row, middle column). **(B)** An example of an array from the guns on complex background condition (gun in middle row, left column). **(C)** An example of an array from the spiders on white background condition (spider in middle row, right column). **(D)** An example of an array from the spiders on complex background condition (spider in lower row, left column). Pictures were collected from the internet (under Creative Common License) and from Pixabay (https://pixabay.com/). Four pictures of guns on complex backgrounds were taken from the International Affective Picture Systems [(IAPS; Lang et al., 2008); Pictures taken from IAPS (gun on complex background condition): 6190, 6,200, 6,210, 6,240]. In the actual experiments, pictures were matched for contrast and luminance. In the complex ontogenetic stimuli condition, pictures of guns, phones and staplers often appeared in people’s hands for ecological validity.

Before beginning the experiment, each participant took part in two practice blocks, each one containing ten trials. These practice blocks were not included in the final analysis. Each trial began with a fixation cross (500 ms), after which a cue specifying the probability of the target type on a subsequent search task (e.g., “phone 90%”/“bird 90%,” “gun 90%”/“spider 90%,” “50% gun phone”/“50% spider bird,” “50% phone gun”/“50% bird spider”; 2,500 ms) appeared. The actual congruency rate between cues and targets was 71%, as in our previous studies (Aue et al., 2013, 2016, 2019; Abado et al., 2020c). Then, another fixation cross appeared (500 ms) followed by a search array consisting of eight pictures of staplers or butterflies and one deviant picture (gun/phone in ontogenetic conditions; spider/bird in phylogenetic conditions; 2,500 ms or until response; see Figure 1 for an example of a trial). Each of the two targets had an equal likelihood of appearing in each one of the nine possible locations. Both targets appeared equally often. On 5% of the trials, no deviant picture appeared, so that the search array consisted of nine pictures of distractors (staplers/butterflies). Overall, there were 360 trials. Participants were instructed to determine as quickly and as accurately as possible the category of the deviant stimuli by pressing the P and Q keys for threat and neutral deviants (counterbalanced) or the SPACE bar to indicate no deviant. The participants performed the task in four blocks of 90 trials each.

After 20 practice trials, before the experiment began, participants were asked to rate the probability of encountering the threatening target throughout the experiment using a visual analogue scale (VAS) ranging from 1 to 100%. This measure reflects participants’ *a-priori* expectancy of encountering the fearful stimulus. Post-experimentally, participants were asked to rate a-posteriori frequencies of occurrence (i.e., how often they thought that they in fact encountered each type of target). Participants were also asked to answer a short post-experimental questionnaire (see Supplementary materials for details).

### Design and analysis

Errors made up 6–9% of all responses in the complex background conditions (*SD*: 3–4%) and 4–5% of all responses in the white background conditions (*SD* = 2–3%). There was no sign for a speed-accuracy tradeoff in any of the conditions (all *p*s > 0.05). Per each participant, individual trials were removed if they were ± 2.5 Z scores larger than the mean RT of each of the four within-subject conditions. This led to the removal of 2.5% of individual trials. Sphericity corrections were applied as needed.

A 3 × 2 × 2 × 2 repeated measures analysis of variance (ANOVA) was conducted, with the within-subject factors *cue* (threatening, neutral, ambiguous) and *target* (threatening, neutral) and the between-subject factors *stimulus type* (phylogenetic, ontogenetic) and *background type* (white, complex).

In addition to the ANOVAs, two regression analyses were performed in order to examine the influence of IU and fear of spiders on attention bias toward threatening targets. Attention bias was calculated by subtracting the mean RT for threatening targets from the mean RT for neutral targets, regardless of the preceding cues, for each participant. A regression analysis was conducted with the 4 questionnaires as independent measures (i.e., fear of spiders and IU as constructs of interest; depression and anxiety were included to make sure that they do not explain additional variance). Regression analyses were performed for each of the between-subject conditions separately. Lastly, split-half reliability analyses were conducted in order to estimate the internal consistency of attention bias toward threat. Analyses were conducted using R (R Core Team, 2022) and the “splithalf” package (Parsons, 2020; for further details, see Supplementary materials). For the design and analyses of reported *a-priori* and a-posteriori frequency estimates and post-experimental questionnaire, see the Supplementary materials.

## Results

### Reaction time analysis

**Figure 2:**
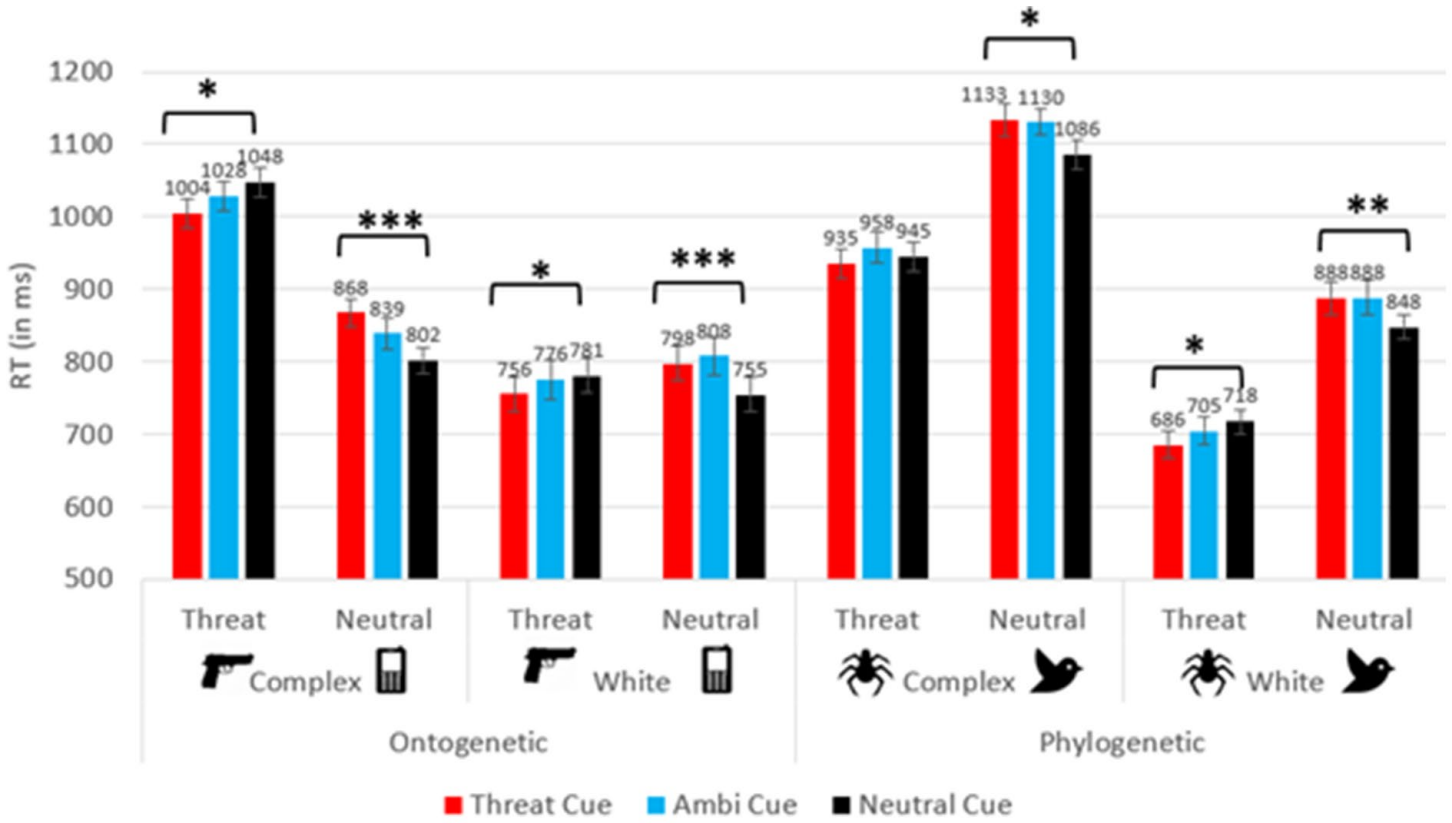
RT for the cue × target interaction in each between-subject condition. Error bars depict standard errors. *p < 0.05, **p < 0.01, ***p < 0.001.

Reaction time (RT) analysis yielded a significant main effect for cue (*F*(2, 277.77) = 7.34, *p* = 0.001, *ƞ^2^p* = 0.050), such that participants responded significantly faster when neutral cues appeared (*M* = 872.77 ms), compared to ambiguous cues (*M* = 891.28 ms; *p* < 0.001) and to threatening cues (*M* = 883.46 ms; *p* = 0.049). No other significant differences between cues emerged (all *p*s > 0.05). No significant interactions arose between cue and any between-subjects factor (all *p*s > 0.05). A main effect of target was found (*F*(1,140) = 18.07, *p* < 0.001, *ƞ^2^p* = 0.114), as participants generally responded faster to threatening stimuli (*M* = 861.69 ms), compared to neutral stimuli (*M* = 903.32 ms). An interaction was revealed between target and stimulus type (*F*(1,140) = 173.75, *p* < 0.001, *ƞ^2^p* = 0.554). Additional interactions emerged between target and background type (*F*(1,140) = 28.18, *p* < 0.001, *ƞ^2^p* = 0.168), as well as a triple interaction between target, background type and stimulus type (*F*(1,140) = 27.59, *p* < 0.001, *ƞ^2^p* = 0.165). Additionally, an interaction between cue and target was observed (*F*(1.91, 263.00) = 27.65, *p* < 0.001, *ƞ^2^p* = 165). No further interaction between cue, target and any of the between-subject factors emerged (all *p*s > 0.05). As seen in Figure 2, the differences between congruent and incongruent trials were larger in ontogenetic groups. Specifically, Cohen’s *d* effect sizes ranged from 0.100 to 0.525 in the phylogenetic conditions and from 0.432 to 0.764 in the ontogenetic conditions (in the phylogenetic-complex background condition *d* = 0.100 for spider target trials and 0.403 for bird target trials; in the phylogenetic-white background condition *d* = 0.434 for spider target trials and 0.525 for bird target trials; in the ontogenetic-complex background condition *d* = 0.448 for gun target trials and 0.745 for phone target trials; in the ontogenetic-white background condition *d* = 0.432 for gun target trials and 0.764 for phone target trials).

Main effects of each between-subjects factor were found (stimulus type: *F*(1,140) = 10.88, *p* = 0.001, *ƞ^2^p* = 0.072; background type: *F*(1,140) = 141.18, *p* < 0.001, *ƞ^2^p* = 0.502), as participants responded faster in ontogenetic conditions (*M* = 855.12 ms) compared to phylogenetic conditions (*M* = 909.89 ms) and in white background conditions (*M* = 783.83 ms) compared to complex background conditions (*M* = 981.18 ms).

To better understand the aforementioned effects and interactions, a 3 × 2 repeated measures ANOVA was conducted on each of the four between-subject factor combinations, with the within-subject factors *cue* (threatening, neutral, ambiguous) and *target* (threatening, neutral). In all four conditions, an interaction of cue and target was revealed (all *p*s < 0.05, except for the phylogenetic-complex condition, in which the congruency effect was not significant: *p* = 0.082; 0.065 ≤ *ƞ^2^p* ≤ *0*.249). In addition, in both background conditions, participants responded faster to spider targets compared to bird targets (complex background condition: *F*(1,37) = 41.36, *p < 0*.001, *ƞ^2^p* = 0.528; spider targets: 945.94 ± 19.95 ms; bird targets: 1,116 ± 18.96 ms); (white background condition: *F*(1,35) = 136.13, *p* < 0.001, *ƞ^2^p* = 0.795; spider targets: 703.16 ± 18.44 ms; bird targets: 874.39 ± 20.48 ms). Participants also responded faster to phone targets (835.92 ± 17.46 ms) compared to gun targets (1,026 ± 18.33 ms; *F*(1,35) = 91.81, *p < 0*.001, *ƞ^2^p* = 0.724) on a complex background. No other effects were found (all *p*s > 0.05).

Planned paired-samples *t*-tests were conducted to analyze the cue × target interaction in each of the four between-subject groups (see Figure 2). The analyses compared between congruent and incongruent conditions, for each type of target on each between-subject level, to examine the influence of expectancy cues on the detection of threatening and neutral targets. Generally, these analyses indicated the presence of congruency effects in all conditions, *except for spider targets on a complex background*. In all other conditions, participants responded faster on congruent trials compared to incongruent trials.

### Questionnaires

Correlational analyses were conducted across conditions to examine associations between the different questionnaires (BDI, STAI – state, IUS-12 and FSQ). A small to medium positive correlation was found between fear of spiders (FSQ) and IU (IUS-12; *p* = 0.010, *r* = 0.213), indicating that higher fear of spiders levels were associated with higher levels of IU.

For the attention bias index, none of the four regression models reached significance (all *p*s > 0.05). However, in the phylogenetic-complex background condition, FSQ reached significance (*β* = 0.412, *p* = 0.021). To better understand the meaning of the association of FSQ with attention bias, two subsequent regressions were conducted, each time with a different dependent variable: absolute RT toward bird targets and absolute RT toward spider targets in the phylogenetic-complex background condition. Results showed a significant relationship between FSQ levels and RT toward birds (*β* = 0.327, *p* = 0.045) but not toward spiders (*β* = −0.273, *p* = 0.097). In other words, the higher the fear of spider levels, the longer it took participants to detect bird targets.

### Reliability

For each between-subject condition, split-half reliability for the difference between RTs toward threatening targets and neutral targets showed moderate to excellent reliability of attention bias for all between-subject conditions (0.87–0.93; Koo and Li, 2016; for further details, see Supplementary materials).

For the design and analyses of reported *a-priori* and a-posteriori frequency estimates and post-experimental questions, see the Supplementary materials.

## General discussion

The current study aimed at examining whether the attention bias previously found toward spiders extends toward ontogenetic threat (i.e., guns). To this aim, ontogenetic and phylogenetic stimuli were directly compared in terms of expectancies and attention. Visual factors were also controlled, as pictures appeared on complex ecological backgrounds or on white sterile backgrounds. Results showed a robust and reliable attention bias toward spiders, as participants detected spider targets faster than bird targets, regardless of the type of background, while no bias toward guns emerged. With regards to expectancy, participants *did not* use the cues to detect spider targets on complex background, thus fully replicating our previous findings (Aue et al., 2013, 2016, 2019). By contrast, in all other conditions, participants *did* use the cues to detect each type of target.

Interestingly, while attention bias toward spiders was shown regardless of fear of spiders, the more participants were afraid of spiders, the longer it took them to detect birds, replicating previous findings (Aue et al., 2013), presumably due to prolonged checking, to make sure that indeed there were no spiders on the screen. Checking behavior is often a characteristic of other disorders, such as obsessive–compulsive disorder (OCD; for a meta-analysis, see Strauss et al., 2020), therefore it would be interesting to develop corresponding paradigms for such disorders. Along the same lines, fear of spiders was further correlated with trait IU. IU has often been linked to OCD (Sarawgi et al., 2013). The current results suggest a link between checking behavior, fear of spiders, and IU. While in the present study no association was found between checking behavior and IU, future studies should more deeply investigate checking behavior and other expressions of cognitive biases as well as their links to trait IU.

In the current study, participants exhibited biased *a-priori* expectancies to encounter spiders only in the complex background condition. This expectancy bias complements the observed attention bias. Specifically, attention bias was found in two forms: first, a general effect toward spiders was observed, as spiders were detected faster than birds, on both background types. This finding is in line with some previous studies, which revealed faster detection of spiders and other types of phylogenetic threat (e.g., Öhman et al., 2001; Waters et al., 2011). Second, in line with our own previous studies, cues were not used in the detection of spiders (on a complex background), even though they were detected faster. Here, attention interacted with expectancy. Together, these two complimentary effects of attentional bias suggest strong and consistent prioritization of spiders. Importantly, the lack of congruency effect seems to be limited to spiders on an ecological background and does not seem to extend to white, unecological backgrounds. Thus, visual factors, especially complex ones, may contribute to participants’ attention deployment to threat – including its immunity to prior expectancies – as the threat may seem more real or imminent.

The current study focused on factors that affect attention bias and on the comparison of attentional allocation for different types of stimuli on different visual backgrounds. However, many studies and theories have been written on why certain stimuli, such as spiders and snakes, receive prioritized processing to begin with. Such studies focus mainly on the origin of fear toward such phobic stimuli and situations. For instance, in his theory about phobias and preparedness, Seligman (1971) argued that phobias of a specific set of “biologically relevant” stimuli (e.g., animals, blood, heights) are due to preparedness to fear such stimuli. Meanwhile, according to this hypothesis, phobias of more modern stimuli are less common because humans are less “prepared” to fear them. However, recent evidence and reviews suggest that humans are not predisposed to *fear and avoid* stimuli such as spiders and snakes, but rather that we are predisposed to generally *detect any type* of potential harm (e.g., many types of different animals, including curvilinear shapes that resemble snakes) and that we gradually habituate to animals that are non-threatening (for a review, see Coelho et al., 2019). Along the same lines, in a series of systematic studies, New et al. (2007) suggest that humans exhibit prioritized processing, or an “animate monitoring bias” toward all types of animals, regardless of their respective threat values, but not to objects, even fast and fatal objects, such as moving cars. Thus, the authors conclude that this bias toward animals exists due to ancestral priorities. Nonetheless, in the current study and in our previous studies, we found attention bias to spiders, which did not extend to other animals, namely birds.

It is important to note that while some studies focus on prioritized processing of animals in general or on spiders in particular, other studies make a distinction between spiders and snakes, as objectively, snakes pose a larger threat than spiders and thus it would make evolutionary sense for snakes to receive more cognitive resources (e.g., Soares and Esteves, 2013; Van Strien et al., 2016; for a review, see Öhman et al., 2012). This finding has also been found in snake-naïve Japanese monkeys, which suggests that attention bias toward snakes may have an evolutionary basis, while attention to spiders may be driven by other top-down factors (Kawai and Koda, 2016) or by socio-cultural learning (Luck et al., 2020).

Other researchers also suggest that fear of spiders does not necessarily make evolutionary sense, as the vast majority of spiders are harmless to humans (e.g., Hauke and Herzig, 2017) and thus extreme fear and avoidance of spiders is not evolutionary adaptive in terms of the trade-off between costs and benefits. According to this argument, fear of spiders is a generalized form of fear of a similar looking animal, which are indeed evolutionary-relevant and potentially more dangerous: scorpions (Landová et al., 2021; Rudolfová et al., 2022). According to this argument, fear of scorpions has been generalized to spiders due to their shared visual similarities. This suggestion is in line with studies demonstrating perceptual interpretation biases toward spiders, as participants with spider fear “detected” spider pictures even when they were in fact pictures of beetles (Becker and Rinck, 2004; see also Ginat-Frolich et al., 2019, for more on fear generalization in spider fear). Of note, in addition to inducing fear, spiders are also often rated as extremely disgusting, even in unselected samples (e.g., Polák et al., 2020). Thus, apart from fear, disgust may also play a prominent role in aversion of spiders.

The current study points to the existence of various moderators of attention bias to various types of threat. These can explain why the current study found prioritize processing of phylogenetic threat, while other studies found different results. These various results could be due to the fact that the current paradigm included expectancy manipulations and was different from other studies. While the current study could not address all moderators of attention bias, they include arousal levels (e.g., Zsido et al., 2019b, 2020), perceived danger that the stimulus induces (Brosch and Sharma, 2005), as well as perceived unpredictability and uncontrollability of the stimulus/situation (e.g., Cao et al., 2014; for a review, see Armfield, 2006). Low-level variables, such as the shape of the stimulus, have also been found to affect the detection of threat (e.g., Van Strien et al., 2016; Givon-Benjio and Okon-Singer, 2022). Individual traits, such as disgust propensity, and sociodemographic variables, such as gender, age, level of education, biology background, have also been found to affect the detection of threat (see Polák et al., 2020, 2022, for more on disgust and fear in the perception of animals in non-clinical sample; for reviews on interactions between bottom-up and top-down factors that affect the perception of and attention toward threat, see Sussman et al., 2016; Abado et al., 2020a; Cinq-Mars et al., 2022, for more details on top-down processes in the processing of threat; see Godwin et al., 2016, for more details on expectancy manipulations in visual search).

Recent studies suggest inconsistent reliability and within-subject differences in popular cognitive tasks (Hedge et al., 2018; Parsons et al., 2019). Specifically, the lack of replicability in the case of attention bias toward threat has led to many debates about the importance of measuring reliability (Rodebaugh et al., 2016). For this reason, half-split reliability analyses for attention bias were also conducted here. Our RT results were further validated by the reliability analysis, which indicated moderate to excellent internal reliability of attention bias, measured as the difference in RT between threatening and neutral targets. In addition, our findings have high levels of external validity, as participants were not pre-selected in terms of fear.

The simultaneous assessment of several biases is in line with the combined cognitive bias hypothesis account, which advocates the integrative study of biases and of their interactions. Such an approach could be more valid as well as more informative regarding the complexities of biased cognitive and emotional processes (for a review, see Everaert and Koster, 2020; for a recent study on the combined cognitive bias hypothesis in adolescence, see Parsons et al., 2021). Furthermore, contemporary studies suggest that different disorders can be characterized by a unique pattern of cognitive biases that is exhibited in each disorder (Richter et al., 2020).

As mentioned earlier, one of this investigation’s main goals was to examine the role of ecological factors in attention bias toward spiders and to externalize previous findings. It is important to note, however, that our task could benefit from even higher levels of ecological validity. Specifically, the task asks participants to detect a deviant picture among eight distractors, where all pictures are of the same size. This task could be made more ecological by presenting participants with a real-life scene and by tracking their eye-movements. Making the task more ecological can lead to more fine-tuned results and to a better understanding of the interaction between expectancy and attention. Additionally, participants can be provided with the real-life likelihood of encountering spiders in different settings (e.g., in the woods, in urban settings), thereby adapting the experiment more into a cognitive training which assists participants in reducing attention bias levels (for more on cognitive trainings and attention bias modification, see McNally, 2019; Shani et al., 2019; Richter et al., 2020).

To the best of our knowledge, this is the first study to show a correlation between fear of spiders and trait IU in an unselected sample. IU is considered a transdiagnostic trait, which is found in many disorders, including generalized anxiety disorder, social anxiety disorder, panic disorder, agoraphobia, OCD, depression, and eating disorders (for a recent meta-analysis, see McEvoy et al., 2019). While IU has been studied extensively in many anxiety as well as other psychiatric disorders, it has not been studied often in specific fears (for reviews, see Carleton, 2012, 2016; Grupe and Nitschke, 2013; Shihata et al., 2016; Rosser, 2019). As the links between trait IU and cognitive biases are also understudied, future investigations should examine the associations between specific fear levels, cognitive biases, and trait IU, in order to form a more holistic view of IU.

A limitation of the current study consists of the type of pictures that were chosen. Specifically, one difference between the types of pictures that were presented is that guns were presented in people’s hands, while pictures of spiders did not include any human body part. While this difference was a part of the experimental manipulation, it may have added a confound, as the presence of human body parts can change how threatening stimuli are processed (e.g., Cao et al., 2014). In the present study, human hands were added to gun pictures in order to make them seem threatening. This is in line with previous observations, which suggest that in order to be perceived as a potential threat, a situation/event needs to be first evaluated as unpredictable, uncontrollable and dangerous (for a review, see Armfield, 2006). While this is usually the case with spiders (e.g., Grill and Haberkamp, 2023), this is not the case with guns or any other object, unless it is actively manipulated by some external force (such as a human being holding it). The findings of Cao et al. (2014) are also in line with the current study, in which pictures of guns were rated as more unpleasant than pleasant, especially in the complex background condition, in which guns were present in human hands. Nonetheless, despite their unpleasantness, no attention bias was found toward guns on either type of background.

These findings could have important clinical implications. For instance, developing a cognitive training procedure which reduces attention bias might reduce fear in the therapeutic context (for a review, see Van Bockstaele et al., 2014; Abado et al., 2020c, for the modification of attention bias using a manipulation of frequencies). Future studies could examine the role of IU in attention bias toward threat, and thus IU targeted attention bias modification procedures could be developed (for IU in CBT, see Dugas et al., 2010; Hebert and Dugas, 2019). Checking behavior may also be related to IU and thus individually tailored and IU targeted treatments may reduce attention bias as well as checking behavior.

To summarize, the current study sought to compare expectancies and attention bias between two types of threatening stimuli, phylogenetic (i.e., spiders) and ontogenetic (i.e., guns), while also taking into account visual factors (i.e., sterile or ecological backgrounds). Whereas attention bias to spiders was found on both backgrounds, no attention bias was found toward guns. Additionally, whereas participants used the cues to detect spiders on a white background, cues were not used to detect spiders on a complex background. Lastly, a small to medium positive correlation was found between the time it took participants to detect birds on a complex background and pre-existing fear of spiders levels. While our results suggest prioritized processing of spiders, the reason for this prioritization, whether evolutionary or socio-cultural, is still unknown.

## Data availability statement

The raw data supporting the conclusions of this article will be made available by the authors, without undue reservation.

## Ethics statement

The studies involving humans were approved by this experiment was approved by the ethics committee of the School of Psychological Sciences at the University of Haifa (approval #341/19). The studies were conducted in accordance with the local legislation and institutional requirements. The participants provided their written informed consent to participate in this study.

## Author contributions

EA, HO-S, and TA developed the experimental paradigm. EA recruited participants and collected and analyzed the data under the supervision of HO-S. EA, TA, and HO-S drafted the manuscripts. All authors contributed to the article and approved the submitted version.

## Funding

This work was supported by the Israel Science Foundation (grant #823/18) awarded to HO-S, as well as funding given to EA from the Budgeting and Planning Committee (Higher Education Council, Education of Ministry) and The Herta & Paul Amir Faculty of Social Sciences, University of Haifa. The funding source had no involvement in the study design, collection, analysis or interpretation of the data, writing the manuscript, or the decision to submit the paper for publication.

## Conflict of interest

The authors declare that the research was conducted in the absence of any commercial or financial relationships that could be construed as a potential conflict of interest.

## Publisher’s note

All claims expressed in this article are solely those of the authors and do not necessarily represent those of their affiliated organizations, or those of the publisher, the editors and the reviewers. Any product that may be evaluated in this article, or claim that may be made by its manufacturer, is not guaranteed or endorsed by the publisher.

## Footnotes

^1^ https://www.prolific.co/
