# Supplementary material for: Spider vs. guns: expectancy and attention biases to phylogenetic threat do not extend to ontogenetic threat
Source: Front Psychol. 2023 Aug 30;14:1232985. doi: 10.3389/fpsyg.2023.1232985 (PMC10498540; doi:10.3389/fpsyg.2023.1232985)
Supplement: Supplementary file 1 [file Data_Sheet_1.docx]

**Supplementary Materials**

# **Method - Post-Experimental Questionnaire**

**Procedure.** Following the experimental paradigm, participants were asked to answer several questions which acted as manipulation checks as well as measurement of explicit knowledge of participants’ awareness of their RT and cue-target congruency rates. There measures included: (a) indicate if they felt, during the experiment, that they reacted faster to a specific target. Possible answers were: “yes, I responded faster to the threatening target”; “yes, I responded faster to the neutral target”, and “no, I responded similarly to both types of targets”. This question reflects explicit awareness of attention bias toward a certain target; (b) rate the probability of threatening targets that they have encountered throughout the experiment (i.e. a-posteriori expectancy, on a VAS that is identical to the one used to measure a-priori expectancy); (c) indicate if they felt that the cues truly predicted the target (using a VAS; 71% of trials were congruent, thereby representing some but not total predictiveness). Lastly, to rule out valence as an intervening factor, participants were presented with 30 random pictures from the experiment (10 from each category – threatening target, neutral target and distractors) and for each picture they were asked to indicate how pleasant and how unpleasant it was perceived, each on a scale from 0-8 (Kron et al., 2013). Every picture appeared for 4 seconds, followed by the two scales.

**Design and Analysis.** In order to examine whether participants over-estimated the appearance of spiders before and/or after the experiment, for both types of expectancies in each condition, one-sample t-tests were conducted, with a test value of 50% (i.e., the true likelihood of encountering either type of target). To analyze the question about the cues’ validity, one-sample t-tests were conducted with a test of value of 71% (i.e., the true predictive value of the cues) for each between-subject group. To analyze the valence ratings of each type of stimulus (ranging from 0-8), three paired t-tests were conducted for each between-subject group. These analyses examined whether each type of picture (threatening target, neutral target, and distractor) was considered significantly more pleasant than unpleasant (or vice-versa).

# **Results**

**Questionnaires**

The mean BDI score for the sample was 13.77±10.62; the mean STAI (state) score was 40.76±10.82; the mean FSQ score was 47.81±30.27 and the mean IUS-12 score was 34.84±8.71.

**Expectancies and Post-Experimental Questionnaire**

1. A-priori and a-posteriori encounter expectancies for each threatening target were measured. Both types of expectancies were similar for all conditions, ranging from 54%-59%. In each between-subject condition, one-sample t-tests revealed that a-posteriorily, participants significantly overestimated having encountered the threatening stimulus (all *p*s < .05). However, a-priorily, participants overestimated the likelihood of encountering the threatening stimulus only in the phylogenetic-complex background condition (*p* = .019).
2. Participants were asked if they felt that they responded faster to a certain target. Results indicate that overall, in each condition, participants were indeed aware of the speed of their responses toward each type of target. Specifically, on each background, most participants (66%-81%) indicated that they detected spiders faster than birds. For ontogenetic-complex background condition, most participants (61%) indicated that they detected phones faster than guns. On the white background, most participants (53%) indicated that they detected guns faster than phones while in reality participants’ RTs were similar for both targets in this condition.
3. Participants were asked to what extent they felt that the cues were predictive. In each of the 4 between-subject groups, one-sample t-tests revealed that participants significantly underestimated the true predictive value of the cues (all *p*s < .001; 41%<*M*<52%).
4. Valence ratings: Generally, results show that threatening targets were rated as more unpleasant than pleasant, while the reverse was true for neutral targets and distractors (see Fig. 1).


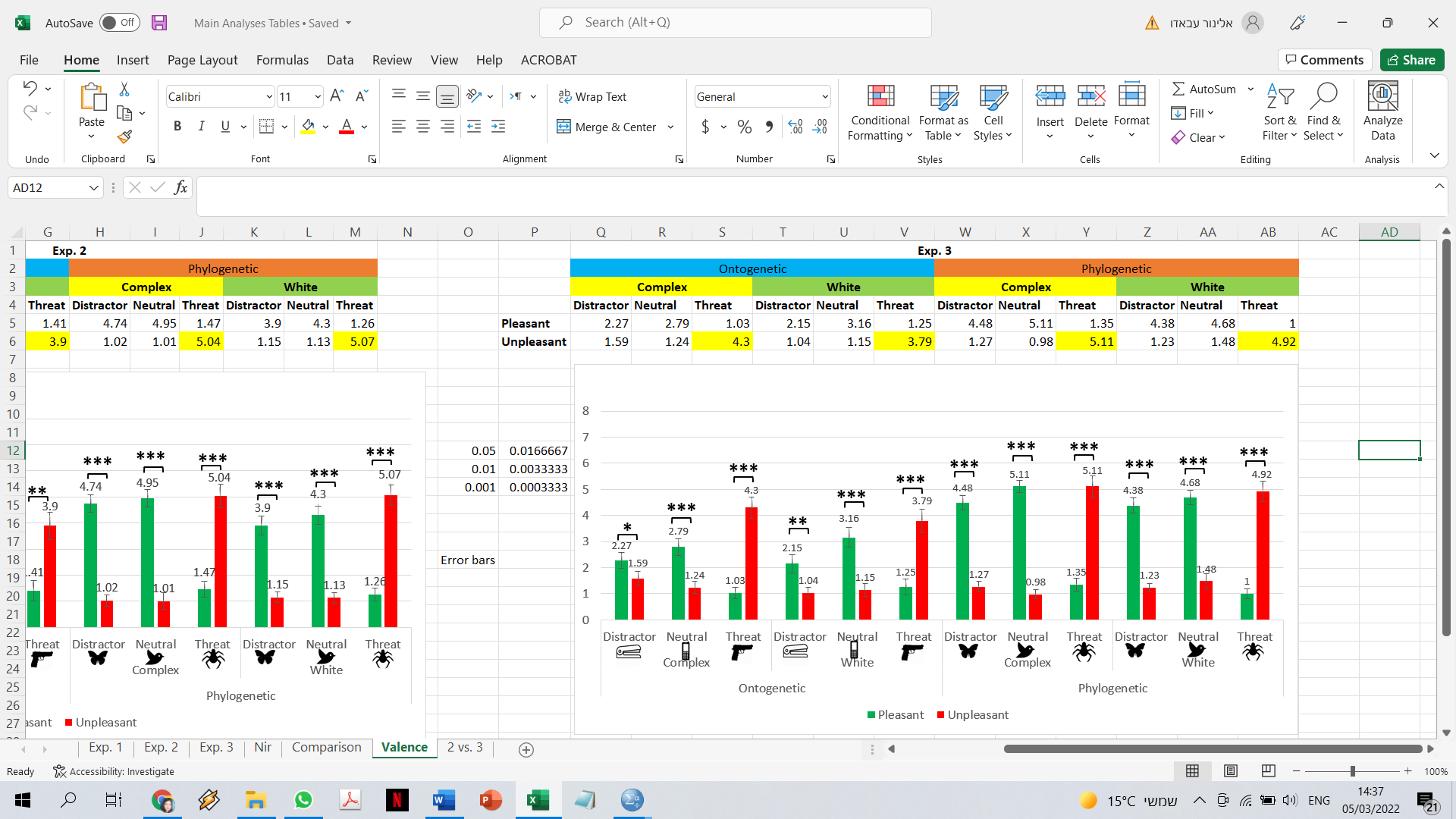


**Fig. 1.** Ratings of picture by scale (pleasant, unpleasant), stimulus group (phylogenetic, ontogenetic), background type (complex, white) and picture type (distractor, neutral target, threatening target). *p < 0.05, **p < .01, ***p < .001.

1. Reported perceived disgust, danger, uncontrollability and unpredictability of spiders were positively correlated with fear of spiders (all *ps* <.001; .661<*r*<.729) and intolerance of uncertainty (disgust and danger were almost significant: *p* = .057, *r* = .159, *p* = .051, *r* = .163, respectively; uncontrollability: *p* = .039, *r* = .172; unpredictability: *p* = .001, *r* = .262). State anxiety levels and depression levels correlated with unpredictability (STAI: *p* = .034, *r* = .177; BDI: *p* = .029, *r* = .181) and almost significantly with uncontrollability (STAI: *p* = .051, *r* = .163; BDI: *p* = .053, *r* = .161).

**Reliability Analyses**

These analyses were conducted on raw data and on correct answers only. Outlier trials (Z score over 2.5 for each participant in each within-subject condition) were excluded. Split-half reliability was measured for the difference between RTs toward threatening targets vs. neutral targets (i.e., the attention bias measure), for each between-subject condition. For each participant, this analysis compares one half of certain trials with the second half. Trials are split randomly using 5,000 permutations (repetitions) and for each half, outcome scores are calculated (“splithalf” package; Parsons, 2020). For every permutation, the correlation coefficient between each half is calculated and then the average of these correlations is taken as the final estimate of reliability.

For the ontogenetic-complex background condition, the Spearman-Brown corrected split-half internal consistency was rSB = 0.90, 95% CI [0.85, 0.94]. For the ontogenetic-white background condition, the Spearman-Brown corrected split-half internal consistency was rSB = 0.87, 95% CI [0.79, 0.93]. For the phylogenetic-complex background condition, the Spearman-Brown corrected split-half internal consistency was rSB = 0.93, 95% CI [0.89, 0.96]. For the phylogenetic-white background condition, the Spearman-Brown corrected split-half internal consistency was rSB = 0.87, 95% CI [0.79, 0.93].

# **Discussion – Post-Experimental Questions**

When asked explicitly about which target elicited the fastest response, most participants correctly identified the target in each condition. However, when asked about the cues’ validity, participants generally underestimated the predictive values of the cues. Thus, while participants exhibited explicit knowledge of their fact reaction toward the targets, they did not explicitly know to what extent they used the cues to detect targets. This discrepancy may be related to the strong effects of exogenous attention driven by the threatening value of spiders, while the endogenous effects of cues may be more subtle.

**References**

Parsons, S. (2020). *splithalf: Robust estimates of split half reliability* (p. 27855 Bytes). figshare. https://doi.org/10.6084/M9.FIGSHARE.11956746.V4
